# Twenty years of antimicrobial resistance control programme in a regional multi hospital institution, with focus on emerging bacteria (VRE and CPE)

**DOI:** 10.1186/2047-2994-1-9

**Published:** 2012-02-13

**Authors:** Sandra Fournier, Christian Brun-Buisson, Vincent Jarlier

**Affiliations:** 1 Direction de la Politique Médicale, Assistance publique-Hôpitaux de Paris (AP-HP), Paris, France; 2 Hôpital Henri Mondor, AP-HP, Créteil, France; 3 UPMC Univ Paris 6 EA1541 Bactériologie-Hygiène 75005 and Hôpital Pitié-Salpêtrière, AP-HP, 75013, Paris, France

**Keywords:** antibiotic resistance control, carbapenemase producing enterobacteria, CPE, vancomycin resistant enterococci, VRE, MRSA, quality programme, healthcare associated infections, HAI

## Abstract

**Background:**

Assistance Publique-Hôpitaux de Paris (APHP), the largest public health care institution in France (38 hospitals, 23,000 beds, serving 11.6 millions inhabitants) launched in 1993 a long term programme to control and survey multidrug resistant bacteria (MDR).

**Findings:**

AP-HP MDR programme consisted in successive waves of actions: bundle measures to survey and control cross transmission of MRSA and extended-spectrum betalactamase producing enterobacteria (ESBL) in 1993, large campaign to promote the use of alcohol-based hand rub solution (ABHRS) in 2001, specific strategy to quickly control the spread of emerging MDR (vancomycin resistant *Enterococcus*, VRE; carbapenemase producing enterobacteria, CPE) in 2006, large campaign to decrease antibiotics consumption in 2006.

Following this programme, the ABHRS consumption dramatically increased, the antibiotic consumption decreased by 10%, the incidence of MRSA, including MRSA bacteraemia, decreased by 2/3, all VRE and CPE events were rapidly controlled. However, the incidence of ESBL, mainly *Klebsiella pneumoniae *and *Escherichia coli*, that remained low and stable until 2003 increased markedly afterwards, justifying adapting our programme in the future.

**Conclusion:**

A sustained and coordinated strategy can lead to control multidrug resistant bacteria at the level of a large multihospital institution.

## Background

Multidrug resistance bacteria (MDR) jeopardizes the quality of care by (a) complicating the treatment of healthcare associated infections (HAI) and (b) increasing the incidence of HAIs (e.g. in the case of methicillin resistant *Staphylococcus *aureus, MRSA). Emergence of vancomycin-resistant enterococci (VRE) and carbapenemase-producing enterobacteria (CPE) is nowadays a major public health concern worldwide [1]. In 1993 Assistance Publique-Hôpitaux de Paris (AP-HP), the largest public health care institution in France, launched a long term programme to survey and control MDR.

## Materials and methods

### Setting

AP-HP is a public health institution administering 38 teaching hospitals (23 acute care and 15 rehabilitation/long-term care hospitals, spread over Paris, suburbs and surrounding counties), totalizing 23,000 beds (10% of all public hospital beds in France) and serving 11.6 millions of inhabitants. AP-HP admits approximately 1 million inpatients per year, employs 19,000 physicians, 18,500 nurses and 29,800 assistant nurses. Local administrators and medical committees manage AP-HP hospitals, but decisions on large investments and medical developments are taken by the central administration. A local infection control team (ICT) is in charge of prevention and surveillance of HAI in each hospital but actions of foremost importance for the whole institution, e.g. MDR control programme, are coordinated centrally by a multidisciplinary infection control team (infectious disease physician, bacteriologist, epidemiologist and nurse)[2].

### MDR control programme

In 1993, AP-HP launched a long-term programme to survey and control MDR. Each step of the programme was implemented gradually in every AP-HP hospitals and supported by a strong commitment of AP-HP central and local administration. This implementation was included as incentive in evaluation process within the institution (quality indicator).

The 1^st ^step (1993) was bundle measures to control cross transmission of MRSA, whose incidence was at that time higher in France than in other European countries, and extended-spectrum ß-lactamase producing enterobacteria (ESBL): identification of MDR carriers by passive and active surveillance, barrier precautions, training and feedback [2].

The 2^nd ^step was a large campaign (2001-2002) promoting the use of alcohol-based hand rub solution (ABHRS). This campaign provided pedagogical material to the local ICT (ready to use slide sets, 200,000 brochures, 14,000 posters). Importantly, AP-HP's General Director urged all administrators, head of departments and chief nurses to support the implementation of the campaign.

The 3rd step (2006) was a specific strategy to quickly control the spread of emerging MDR (vancomycin resistant enterococci, VRE; carbapenemase producing enterobacteria, CPE). Indeed, from August 2004 to December 2005, the monthly number of VRE cases increased significantly [3] and the 1st outbreak of CPE [4] occurred in AP-HP hospitals. In response to this worrying situation, an institutional "emerging MDR programme" was designed: (a) quickly reporting every new VRE/CPE case (defined as infected or colonized patient) to the AP-HP central infection control team (CICT), (b) stopping transfers to other units of the hospital or to other hospitals of the cases and of the contact patients, defined as any patient hospitalized in the same unit during the same period of time as a VRE/CPE case, (c) screening contact patients for VRE/CPE carriage (rectal swabbing) extended to those already transferred from the involved unit, and maintained this screening until the outbreak was controlled, i.e. after all VRE/CPE patients have been discharged and after a period of at least three months without new case, (d) identifying discharged VRE/CPE cases and contact patients when readmitted and (e) cohorting patients in 3 distinct areas with dedicated nursing staffs: "VRE/CPE case patients" section, "Contact patients" section, "VRE/CPE-free patients" section for newly admitted patients with no previous contact with the case patients [4]. To stimulate the efforts made by the local infection control teams and administrators, the CICT (a) followed the number of new VRE/CPE cases, and difficulties in programme implementation, (b) visited regularly the hospitals to help the local teams in applying the programme and (c) regularly disseminated results within hospitals and central administration.

In 2008, based on the analysis of the 1^st ^VRE/CPE events [5], ICTs were advised to screen every patient transferred from a foreign hospital for VRE/CPE.

Finally, a 4 years lasting campaign was launched in 2006 to decrease antibiotics consumption and, consequently, the selection pressure on MDR: (a) identifying a physician in each hospital as "antibiotic referent" in charge of implementing antibiotic policy, (b) providing teaching material to these referents (slide sets, 40,000 brochures, 15,000 posters), (c) stimulation by the General Director (see above). This campaign was based on 10 teasing messages: treat only bacterial infections, know when to say "no" to antibiotics, know when to stop antibiotics, treat only infection and no colonization, use antibiotics wisely, know to say "no" to antibiotic associations, re-evaluate antibiotic prescription after 2 days, prevent infections, limit invasive devices, prevent cross transmission.

## Results

**Figure 1:**
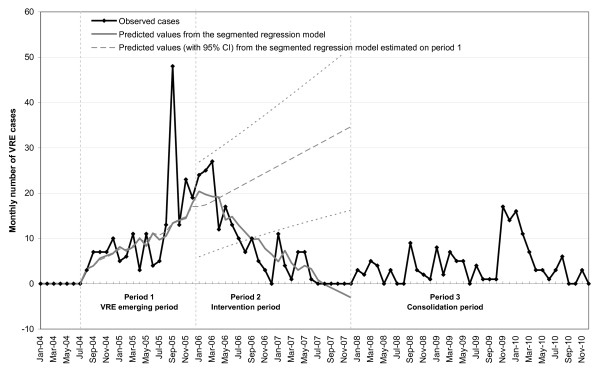
**Observed cases and predicted values (from segmented regression model) of monthly vancomycin-resistant *Enterococcus *(VRE) epidemic cases (infections and colonizations) in the Assistance Publique-Hôpitaux de Paris before (period 1) and after implementation of the institutional VRE infection control programme (periods 2 and 3)**. In period 1, measures to control cross transmission of endemic MDR such as MRSA were applied, in period 2, a specific strategy to control the spread of emerging MDR was instituted. In period 3, this strategy was maintained as routine.

The main results obtained in AP-HP through the programme were as follows:

(a) Global ABHRS consumption increased from 2 to 30 litres per 1,000 hospital days (HD) between 2001 and 2010,

(b) Global antibiotic consumption decreased from 573 to 515 defined daily doses/1,000 HD between 2005 and 2010,

(c) All VRE and CPE events were rapidly controlled. Indeed, after implementation of the specific measures in 2006, a progressive and significant decrease in the number of VRE cases was observed in AP-HP, contrasting with the continuous increase that prevailed in 2004-05, when only guidelines aiming at controlling cross transmission of endemic multidrug resistant bacteria such as MRSA were used (Figure 1).

The follow up of CPE shows that 63 events (1 event = 1 index case with or without secondary cases) occurred between 2004 and 2011, resulting in 107 cases of infections or colonisations. Fifty-three of the events did not lead to secondary cases whereas the ten others led to outbreaks, totalizing 44 secondary cases (1-12 cases per outbreak) [6]. The mean number of cases identified by CPE event, including the index case, decreased from 4.5 to 1.5 between 2004 and 2011, emphasizing efficacy of the specific control measures [4,6]. Among the 63 events, 88% involved patients transferred from foreign hospitals. *Klebsiella pneumoniae *was isolated in 43 events (including 8 outbreaks), *Escherichia coli *in 18 events (including 2 outbreaks), *Enterobacter cloacae *in 3 events, *Citrobacter freundii *and *Enterobacter aerogenes *in 1 event each. Two species of enterobacteria were isolated in 3 episodes. The carbapenemases involved in the 63 events were OXA-48 (51%), KPC (35%), VIM (6%), NDM-1 (8%).

**Figure 2:**
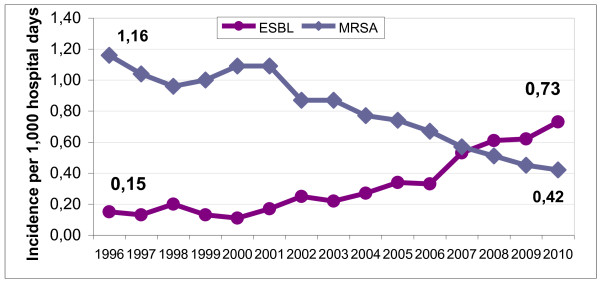
**Change in the incidence per 1,000 hospital days (HD) of methicillin-resistant *Staphylococcus aureus *(MRSA) and extended-spectrum ß-lactamase producing enterobacteria (ESBL) in acute care hospitals**.

(d) The incidence of MRSA, including MRSA bacteraemia, decreased by 2/3 between 1993 and 2010 (Figure 2) [2],

(e) Finally, in contrast to the evolution of the MRSA incidence seen above, the ESBL incidence, involving mainly *K. pneumoniae *and *E. coli*, was contained at low rates (around 0.15 case per 1,000 HD) between 1993 and 2003, but markedly increased afterwards up to 0.73 per 1,000 HD (Figure 2). The proportions of *K. pneumoniae *and *E. coli *were 10% and 60% in 1996 and 50% and 20% in 2010, respectively. This increase of ESBL incidence justifies to adapt our programme, particularly concerning antibiotic policy, hand hygiene and excreta management policy.

## Conclusion

The AP-HP experience shows that a multifaceted, sustained and coordinated strategy can lead to control MDR, including the most resistant emerging ones, at the level of a large regional multihospital institution. Efforts should be maintained to continue to decrease MRSA rates and to contain emergence of CPE and VRE and reinforced to stop the progression of ESBL.

## Competing interests

None of the authors have any competing interests in respect to this manuscript.

## Authors' contributions

SF participated in the design of the programme, performed analysis and interpretation of the data, and wrote the manuscript. CB participated in the design of the programme. VJ participated in the design of the programme, in analysis of the data and corrected the manuscript. All authors read and approved the final manuscript.
